# Genome-wide case-only analysis of gene-gene interactions with known Parkinson’s disease risk variants reveals link between *LRRK2* and *SYT10*

**DOI:** 10.1038/s41531-023-00550-9

**Published:** 2023-06-29

**Authors:** Milda Aleknonytė-Resch, Joanne Trinh, Hampton Leonard, Sylvie Delcambre, Elsa Leitão, Dongbing Lai, Semra Smajić, Avi Orr-Urtreger, Avner Thaler, Cornelis Blauwendraat, Arunabh Sharma, Mary B. Makarious, Jonggeol Jeff Kim, Julie Lake, Pegah Rahmati, Sandra Freitag-Wolf, Philip Seibler, Tatiana Foroud, Andrew B. Singleton, The International Parkinson Disease Genomics Consortium, Anne Grünewald, Frank Kaiser, Christine Klein, Michael Krawczak, Astrid Dempfle

**Affiliations:** 1 Institute of Medical Informatics and Statistics, Kiel University, Kiel, Germany; 2 Department of Computer Science, Kiel University, Kiel, Germany; 3 Institute of Neurogenetics, University of Lübeck, University Medical Center Schleswig-Holstein, Campus Lübeck, Germany; 4 Laboratory of Neurogenetics, National Institute on Aging, National Institutes of Health, Bethesda, MD USA; 5 Data Tecnica International LLC, Glen Echo, MD USA; 6 Center for Alzheimer’s and Related Dementias, National Institute on Aging, Bethesda, MD USA; 7 Molecular and Functional Neurobiology Group, Luxembourg Centre for Systems Biomedicine, Esch-sur-Alzette, Luxembourg; 8 Institute of Human Genetics, University Hospital Essen, Essen, Germany; 9 Department of Medical and Molecular Genetics, Indiana University School of Medicine, Indianapolis, IN USA; 10 Neurological Institute, Tel Aviv Sourasky Medical Center, Sackler Faculty of Medicine and Sagol School of Neuroscience, Tel Aviv University, Tel Aviv Israel; 11 Department of Clinical and Movement Neurosciences, University College London Queen Square Institute of Neurology, London, UK; 12 UCL Movement Disorders Centre, University College London, London, UK

**Keywords:** Parkinson's disease, Genomics, Epidemiology

## Abstract

The effects of one genetic factor upon Parkinson’s disease (PD) risk may be modified by other genetic factors. Such gene-gene interaction (G×G) could explain some of the ‘missing heritability’ of PD and the reduced penetrance of known PD risk variants. Using the largest single nucleotide polymorphism (SNP) genotype data set currently available for PD (18,688 patients), provided by the International Parkinson’s Disease Genomics Consortium, we studied G×G with a case-only (CO) design. To this end, we paired each of 90 SNPs previously reported to be associated with PD with one of 7.8 million quality-controlled SNPs from a genome-wide panel. Support of any putative G×G interactions found was sought by the analysis of independent genotype-phenotype and experimental data. A total of 116 significant pairwise SNP genotype associations were identified in PD cases, pointing towards G×G. The most prominent associations involved a region on chromosome 12q containing SNP rs76904798, which is a non-coding variant of the *LRRK2* gene. It yielded the lowest interaction *p*-value overall with SNP rs1007709 in the promoter region of the *SYT10* gene (interaction OR = 1.80, 95% CI: 1.65–1.95, *p* = 2.7 × 10^−43^). SNPs around *SYT10* were also associated with the age-at-onset of PD in an independent cohort of carriers of *LRRK2* mutation p.G2019S. Moreover, *SYT10* gene expression during neuronal development was found to differ between cells from affected and non-affected p.G2019S carriers. G×G interaction on PD risk, involving the *LRRK2* and *SYT10* gene regions, is biologically plausible owing to the known link between PD and *LRRK2*, its involvement in neural plasticity, and the contribution of *SYT10* to the exocytosis of secretory vesicles in neurons.

## Introduction

Parkinson’s disease (PD), the most common movement disorder world-wide, represents a ‘complex disease’ because multiple genetic and non-genetic factors play a role in its etiology^1^. The population prevalence of PD in developed countries is ~0.3% in early and midlife, rising to 1–2% after the age of 60^2^.

Genome-wide association studies (GWAS) of single nucleotide polymorphisms (SNPs) have identified genes (or genetic regions) associated with various complex diseases^3^, including PD. In fact, the most recent GWAS for PD^4^ identified 90 genome-wide significant disease associations with SNPs from 78 genomic regions. These SNPs account for 16% to 36% of the heritability of PD risk, dependent upon population.

The extent to which SNPs alone can explain the heritability of complex diseases is limited^5^. Although there are ‘PD genes’ with strong effects, such as *LRRK2*, *SNCA* and *VPS35*^6^, their variation contributes little to PD incidence at the population level. Genetic variants associated with idiopathic PD, on the other hand, usually have only small effects upon PD risk, not least because their contribution is likely modified by other genetic and environmental factors. Such statistical gene-gene (G×G) and gene-environment interactions (G×E) have been thought even to represent hallmarks of common complex diseases^7^ so that, although this view is still controversial^8^, a comprehensive search for G×G in PD is warranted.

The meaning of the word ‘interaction’ depends upon the context in which it is being used, either as a biological or as a statistical term^9,10^. Biological interaction usually refers to two factors that are physically or chemically interrelated, or that affect the same disease-relevant biological pathway^11^. Statistical interaction, by contrast, is tantamount to ‘effect modification’, meaning that the disease risk difference associated with one factor on a certain scale depends upon the presence or absence of the other risk factor^12^. In the following, we will focus upon statistical interaction on the logit scale, i.e. we shall deal with departures from the multiplicity of odds ratios. Statistical interaction is generally hoped to point towards biological interaction knowing, however, that the two need not necessarily coincide^13^.

The case-only (CO) design is a statistically powerful approach to studying statistical interaction. It has two main advantages over the case-control (CC) design in that it (i) obviates the need for controls and (ii) achieves greater statistical power than CC with the same number of cases and, of course, zero controls^14^. However, these advantages come at the price of requiring that the two risk factors of interest are uncorrelated in the general population^15^. While the plausibility of such population-level lack of correlation is usually easy to establish for G×E, SNP genotypes can be associated with one another for many reasons, including linkage disequilibrium, cryptic relatedness and genotyping batch effects. However, as we have demonstrated before^16^, such possible limitations of the CO design can be reduced (i) by considering only pairs of SNPs on different chromosome arms in G×G searches and (ii) by relying upon the meta-analysis of center-wise results of such searches.

Previous studies of G×G interaction in PD were mainly focused upon specific genomic regions and either concluded that interaction was lacking^17^, or addressed biological rather than statistical interaction^18,19^. Possible exceptions included a report of putative multi-locus SNP interactions in PD-associated genes^20^ and two focused searches for genetic modifiers of PD risk among *LRRK2* mutation carriers^21,22^, which identified some G×G interaction candidates as well. However, all studies undertaken so far used a CC design and included <2000 subjects.

Here, we report the results of a comprehensive search for G×G interaction in PD, using a CO design. Our study was carried out using the largest SNP genotype data set currently available for PD, comprising >18,000 cases collated by the International Parkinson’s Disease Genomics Consortium (IPDGC). We also sought additional lines of evidence for the plausibility of any statistically significant G×G interaction signals in the IPDGC data, using independent PD genotype and age-at-onset data provided by the Michael J. Fox Foundation LRRK2 Consortium and the Tel Aviv Sourasky Medical Center as well as various in vitro experimental data.

## Results

### Primary G×G search

Our genome-wide search identified 116 significant pairwise SNP genotype associations in all IPDGC cases combined (*p* < 5.6 × 10^−10^; Supplementary Table 1). All associations included either rs76904798 on chromosome 12q or rs76949143 on chromosome 7q as the mandatory main effect (ME) SNP. These associations involved a dominant model for the ME SNP and a dominant or an additive model for the potentially interacting SNP. No significant associations were found in the subgroup of early onset PD cases.

The most convincing associations, by far, were observed between ME SNP rs76904798 on chromosome 12q and three non-ME SNPs on chromosome 12p, namely rs1007709 (interaction OR = 1.80, *p* = 2.7 × 10^−43^), rs117561021 (OR = 3.03, *p* = 2.0 × 10^−35^) and rs140305553 (OR = 3.00, *p* = 8.1 × 10^−35^). For all three associations, an additive model for the non-ME SNP yielded a lower *p*-value than a dominant model, while a dominant model for the non-ME SNPs yielded slightly higher ORs. Genotype counts between ME SNP rs76904798 and rs1007709 showed that carriers of at least one minor allele of both SNPs were strongly overrepresented among cases (Table 1). A comprehensive list of genotype counts obtained for combinations of ME SNP rs76904798 and one of the 12p SNPs studied is provided in Supplementary Table 2. Significant genotype associations involving ME SNP rs76949143 on chromosome 7q were characterized by *p*-values that were more than seven orders of magnitude larger. Moreover, rs76949143 is located in a pseudogene and all the associated SNPs were intergenic. In contrast, according to Ensembl^23^, rs76904798 is a non-coding variant that tags a linkage disequilibrium block of 30,566 base pairs (12:40592225 to 12:40622790, GRCh37) including antisense gene *AC079630.4* and protein-coding gene *LRRK2*. SNP rs76904798 is located in the 5’ region of the *LRRK2* gene, variation of which is one of the most prominent risk factors for both monogenic and idiopathic PD. Therefore, we concentrated our follow-up of the statistical evidence for G×G upon ME SNP rs76904798.

**Table 1:** G×G on PD risk of SNPs rs76904798 and rs1007709.

		rs76904798	rs76904798	rs76904798	Total
		CC	CT	TT	
rs1007709	TT	*7364 (7078.32)*	**2341 (2581.00)**	**207 (252.68)**	9912
rs1007709	TG	**1581 (1830.99)**	*876 (667.64)*	*107 (65.36)*	2564
rs1007709	GG	**75 (110.68)**	*72 (40.36)*	*8 (3.95)*	155
	Total	9020	3589	322	12631

Given are the observed genotype counts of cases in all centers combined. Values in parentheses are the genotype counts expected if SNP genotypes were uncorrelated within each group of cases. Values in bold indicate underrepresented outcomes (observed < expected) and values in italic indicate overrepresented outcomes (observed > expected).

**Fig. 1:**
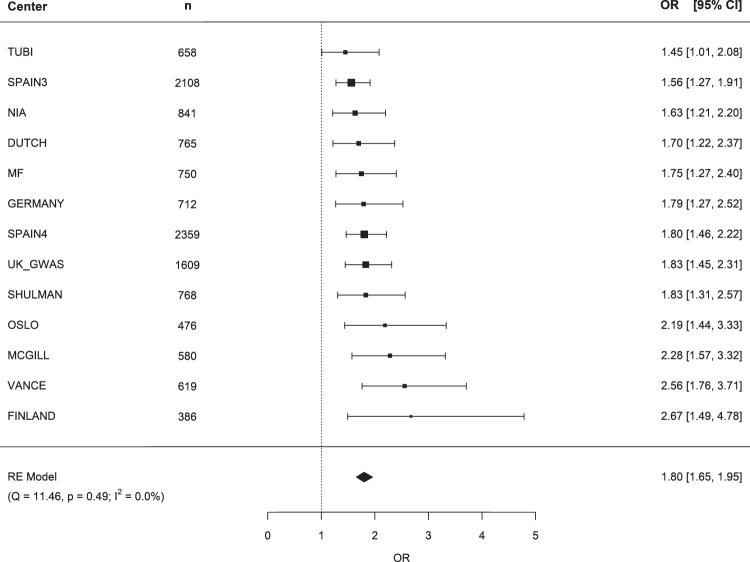
Meta-analysis of the genotypic association, in PD cases, between SNPs rs76904798 and rs1007709. RE random effects, Q Cochran’s Q, *p p*-value of a *χ*^2^ test, *I*^2^ I^2^ statistic of heterogeneity, OR odds ratio, 95%CI 95% confidence interval.

**Fig. 2:**
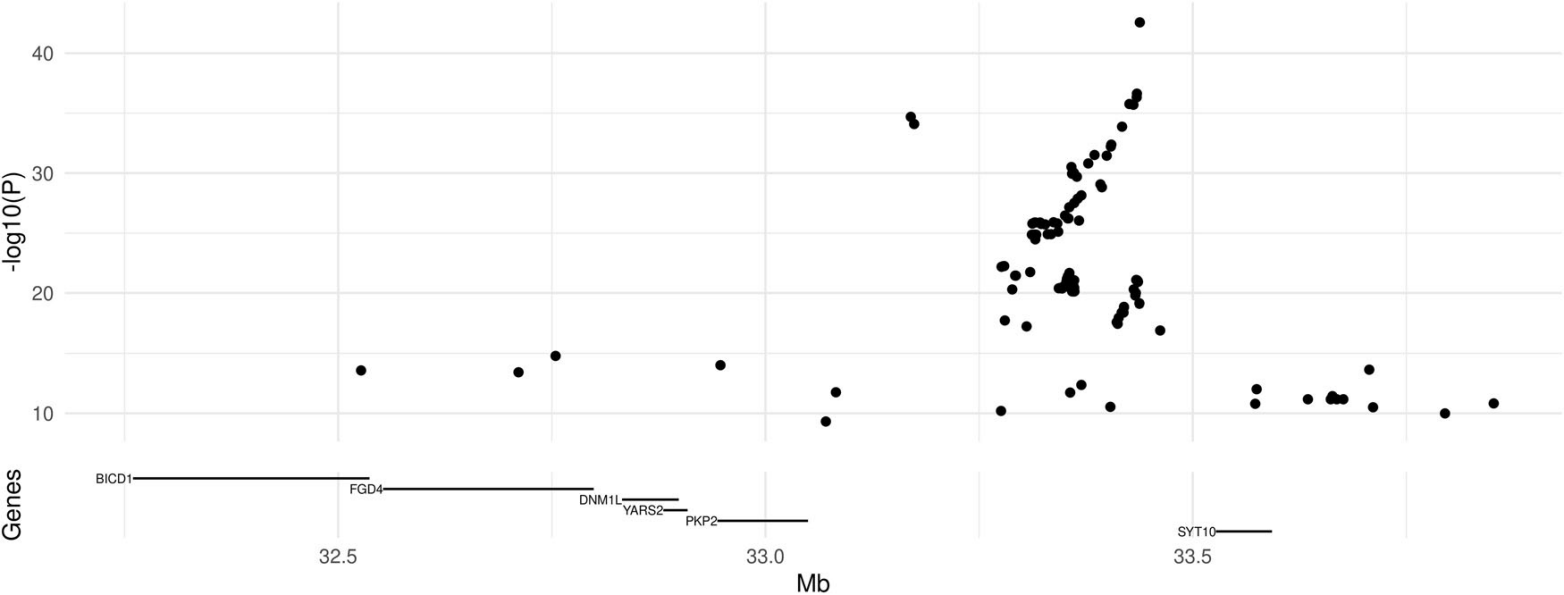
Locus zoom plot of chromosome 12p SNPs associated, in PD cases, with ME SNP rs76904798 (12q). Top panel: log10-transformed *p*-value from meta-analysis of the SNPs from 12:32250000 to 12:34000000 and ME SNP rs76904798; bottom panel: location of protein-coding genes in the region.

The strongest evidence for a genotypic association with rs76904798 was obtained for non-ME SNP rs1007709. Notably, all center-wise interaction ORs for this SNP pair were found to be significantly larger than unity (Fig. 1). The OR estimates ranged from 1.45 (95%CI: [1.01, 2.08]) in Tübingen (TUBI), Germany, to 2.67 (95%CI: [1.49, 1.95]) in Finland. SNP rs1007709 does not belong to any linkage disequilibrium block, according to the 1000 Genomes reference data. Within 10,000 bp on either side, the highest *r*^2^ value with rs1007709 equals 0.01 (for rs182764128, just 10 bp away). Regional locus zoom plots revealed that all chromosome 12p SNPs that exhibited a significant genotypic association, in PD cases, with rs76904798 mapped closely to the *SYT10* gene (Fig. 2).

We also investigated G×G between well-known PD mutation p.G2019S in the *LRRK2* gene (equivalent to SNP rs34637584) and both, 90 previously reported PD risk SNPs and SNPs from a 1 Mb region on either side of *SYT10*. Among IIPDGC cases, p.G2019S had a very low frequency of 0.007, and only few centers reported enough mutation carriers to allow sensible inclusion in the meta-analysis. This notwithstanding, several SNPs near *SYT10* showed a nominally significant association with p.G2019S in IPDGC patients (Supplementary Table 3) while this was not the case for any of the 90 previously reported PD risk SNPs. There was no evidence for an interaction effect between either p.G2019S or ME SNP rs76904798 and rs1007709 on age of onset in PD cases (Supplementary Table 4). An overview of the five pairwise SNP genotype associations with the lowest *p*-values for each ME SNP of interest can be found in Supplementary Table 5. None of the association analyses where both SNPs were part of the set of 90 previously reported PD-associated SNPs reached statistical significance.

### Independent support of potential G×G interactions

A total of 96 SNPs from the 99 SNPs identified in the *SYT10* region had sufficient data to carry out the age-at-onset analysis. Some 34 of the 96 analyzed SNPs from the *SYT10* region showed a nominally significant influence (*p* < 0.05) upon age-at-onset of PD in p.G2019S carriers in the independent *LRRK2* family cohort (Supplementary Table 6). Hazard ratios for the effective alleles of these SNPs (i.e. alleles predisposing to later age-at-onset) ranged from 0.77 to 0.81. In the same vein as the CO design, this age-at-onset association among *LRRK2* mutation carriers points towards an interaction between *LRRK2* and *SYT10*, provided that *SYT10* genotypes are not associated per se with age-at-onset of PD in p.G2019S non-carriers. As far as we are aware, no evidence for such an association has been reported in the scientific literature yet.

### Statistical Power

The statistical power of the CO design of G×G analysis was found to differ widely between the different scenarios considered. With only 2000 cases, the interaction OR must exceed 1.90 for a study to achieve ≥80% power, if MAF = 0.2 for both SNPs, and must exceed 2.95, if MAF = 0.05 (Supplementary Fig. 1a). With a sample size of 18,688 cases like the present study, the interaction OR must be >1.25 for MAF = 0.2, and >1.55 for MAF = 0.05, to achieve ≥80% power (Supplementary Fig. 1b). The CO design provided consistently greater statistical power than a CC design for all scenarios considered.

### Post-hoc assessment of SNP associations in controls (1000 Genomes)

Since the validity of the CO design of G×G analysis hinges upon the population-level independence of the genotypes under study, we investigated SNPs in the centromeric region of chromosome 12 for long-range linkage disequilibrium (LD) in the 1000 Genomes EUR superpopulation. In support of our independence presumption, r² between rs76904798 and SNPs from the *SYT10* region was found to be much smaller in the EUR controls (Supplementary Fig. 2a) than in the PD cases. Furthermore, no systematic increase in D’ could be seen around rs1007709 (Supplementary Fig. 2b). More specifically, for G×G lead SNP rs1007709, r^2^ (D’) was found to be as low as 0.05 (0.22) in the EUR data.

### Experimental results

**Fig. 3:**
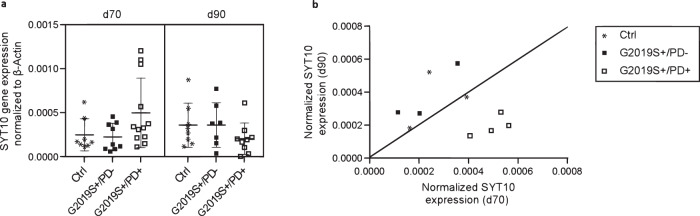
SYT10 expression in iPSC-derived midbrain neurons at days 70 (d70) and 90 (d90) of differentiation. **a** normalized expression by group; Ctrl: controls, G2019S +: heterozygosity for *LRRK2* mutation p.G2019S, PD+ (PD-): affected (unaffected) by PD. The center line denotes the median and the boundaries of the whiskers are based on the 1.5 interquartile range. **b** Average intra-individual SYT10 gene expression at d70 vs d90.

In order to investigate the joint role of *LRRK2* and *SYT10* in PD etiology experimentally, *SYT10* gene expression was measured at days 70 (d70) and 90 (d90) of the differentiation of induced pluripotent stem cells (iPSCs) from four PD patients heterozygous for *LRRK2* mutation p.G2019S (G2019S + /PD + ), three unaffected carriers (G2019S + /PD-) and three controls (G2019S-/PD-). While a trend towards higher *SYT10* expression levels on d70 than d90 was observed for G2019S + /PD + , the opposite was observed for G2019S + /PD- (Fig. 3a). Controls were characterized by similar expression levels at the two time points. When individual-specific measurements were averaged over replicates, all four G2019S+ patients showed downregulation of *SYT10* expression (i.e. mean d70 > d90) and all three unaffected p.G2019S carriers showed upregulation (mean d70 < d90; Fig. 3b; *post-hoc* two-tailed Fisher test *p* = 0.03).

**Fig. 4:**
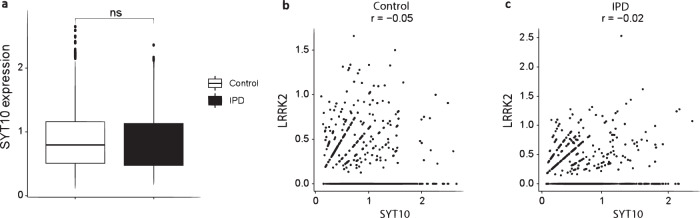
*SYT10* expression in SYT10+ post-mortem midbrain neurons from IPD patients and controls. **a** Box plot of single neuron-level gene expression. The center line denotes the median, the bounds of the box are drawn from the first to the third quartiles and the boundaries of the whiskers are based on the 1.5 interquartile range. **b** Lack of correlation with *LRRK2* expression (log normalized data) in controls (**b**) and IPD neurons (**c**). ns non-significant (Wilcoxon test), r Pearson correlation coefficient.

Inspection of previously published single-cell expression data of post-mortem *SYT10*-positive midbrain neurons suggests that this discrepant expression pattern is specific to p.G2019S carriers. *LRRK2* was expressed in 35% of control neurons (50% of dopaminergic neurons), and in 40% of IPD neurons (54% dopaminergic neurons). *SYT10* was expressed in 22% of control neurons (0% dopaminergic neurons) and in 19% of IPD neurons (7% dopaminergic neurons). No significant difference in *SYT10* expression was observed between idiopathic PD patients and controls (Fig. 4a). Moreover, the expression levels of *SYT10* and *LRRK2* were not correlated in either group (Fig. 4b, c), or in ‘double positive’ neurons alone (Supplementary Material Figs. 3 and 4).

### Regulatory potential of SYT10 SNPs

**Fig. 5:**
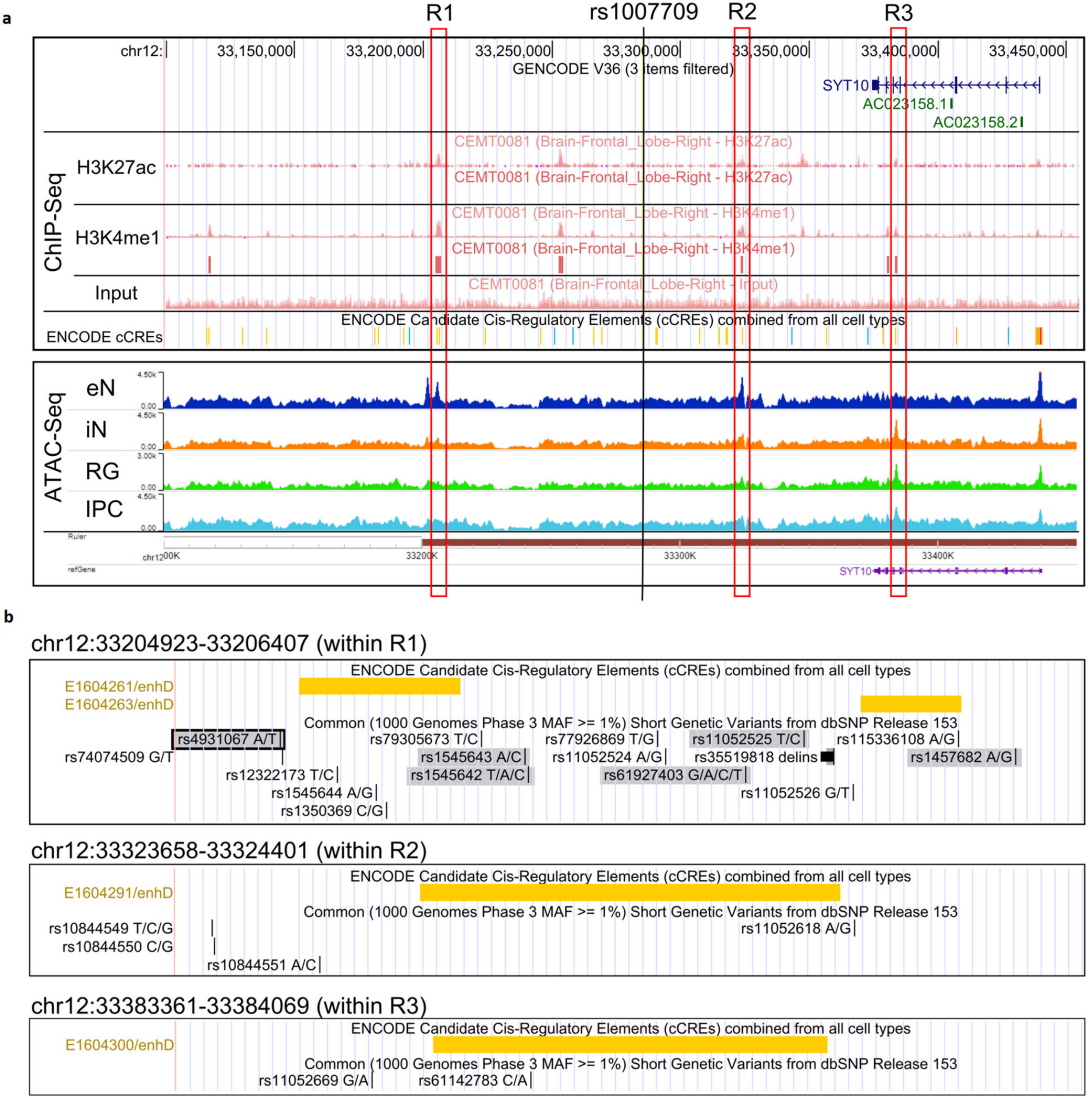
Potential regulatory regions around rs1007709. **a** ChIP-Seq and ATAC-Seq tracks from human brain-related samples. **b** View of regions R1-R3 overlapping with ENCODE cis-regulators elements. SNPs putatively interacting with rs76904798 near *LRRK2* are highlighted in gray. Annotated regulatory SNPs are boxed.

Some 88 of the 99 target SNPs in the *SYT10* gene region were found to represent neuronal eQTLs for *SYT10* (all with a positive normalized effect). Eight SNPs are known regulatory variants and four SNPs overlap, or are located <50 bp away from, a *cis*-regulatory element with distal enhancer-like signatures (Supplementary Table 7). We also investigated publicly available histone modification and chromatin accessibility data from brain-related samples and identified three regions (R1-R3) with H3K27ac and H3K4me1 ChIP-Seq peaks characteristic of enhancers that are located <100 kb from rs1007709 (Fig. 5a, top panel). These three regions also include known ENCODE *cis*-regulatory elements with distal enhancer-like signatures (Fig. 5). In addition, ATAC-Seq data analysis revealed that R1 and R2 are open-chromatin regions in excitatory neurons (eN), but not in interneurons (iN), radial glia (RG) or intermediary progenitor cells (IPC), whereas the chromatin is inaccessible in excitatory neurons only in R3 (Fig. 5a, bottom panel). Interestingly, five of the common SNPs in R1 are GWAS SNPs, and one of them is located <50 bp from a *cis*-regulatory element with distal enhancer-like signatures, also described as a regulatory region variant before (Fig. 5b, Supplementary Table 8). Taken together, these data suggest that regions R1 to R3 potentially act as *SYT10* enhancers (particularly R1), and that local SNPs are plausible candidates for a modulation of the enhancer-promoter interaction and, thus, of *SYT10* gene expression.

## Discussion

We applied the statistically powerful CO design to the largest set of SNP genotype data available from PD cases to date, collated by the IPDGC, to search for hints at G×G interactions upon disease risk. By far the strongest evidence for such interaction was obtained for SNPs in the *LRRK2* and *SYT10* gene regions on chromosome 12, located some 7 Mb apart on either side of the centromeric region, devoid of SNPs. The lowest overall *p*-value (2.7 × 10^−43^, OR = 1.80) was obtained for the genotypic association, in PD cases, between rs76904798, a non-coding variant in the 5’ region of the *LRRK2* gene, and rs1007709, in close proximity to *SYT10*.

Since it cannot be excluded that case-specific SNP-SNP associations such as these are only partially due to statistical G×G interaction, we sought independent lines of evidence for their plausibility. Additional support for an etiological link between the two genomic regions was provided by data from *LRRK2* p.G2019S mutation carriers among whom SNPs in the *SYT10* gene region were associated with the age-at-onset of PD. Finally, experimental data also supported a functional relationship between the two genes in that the course of *SYT10* gene expression during neuronal development in vitro was found to differ between cells from p.G2019S carriers either affected or not affected by PD. Unfortunately, no samples from PD cases lacking the p.G2019S mutation were available to us so that a full resolution of the interaction upon PD risk between *SYT10* gene expression and p.G2019S carriership was beyond the scope of our study. This notwithstanding, together with the enrichment of the *SYT10* gene region with SNPs that act as cis-eQTLs, our results suggest that genotype-specific regulation of *SYT10* may be one of the factors modifying the effects of *LRRK2* variation on PD risk and, to some extent, may thus explain the known reduced penetrance of p.G2019S.

At the genome-wide level, analysis of G×G is computationally challenging because of the large number of pair-wise SNP combinations to consider. Moreover, the large number of hypothesis tests involved requires adjustment of the nominal significance level to an extent that may erode the statistical power of samples as large even as the IPDGC data set used in our study (for a detailed power calculation, see Supplementary Material). We tried to alleviate this problem by focusing upon the most plausible interaction candidates, namely pairs in which at least one SNP had a known ME on disease risk. This strategy ensured good statistical power ( > 80%) with the IPDGC sample even for interaction ORs as small as 1.25 (at moderate minor allele frequency, MAF) or 1.5 (at low MAF; see Supplementary Material). However, our strategy also implied that interactions between SNPs with minor or no ME would have been systematically overlooked. Moreover, SNPs with low MAF are difficult to impute in genomic regions with an ME^24^, and such SNPs may not generate enough carriers of the minor alleles for the logistic regression analysis to converge. Finally, we excluded interaction candidate pairs in which both SNPs were located on the same chromosome arm. Although this restriction greatly reduced the risk of population-level SNP associations jeopardizing the validity of the CO design of G×G analysis^16^, the approach added further to the sources of potential power loss in our study.

Despite the above limitations, we detected a pair of genetic loci with multiple lines of evidence for an interaction on PD risk. The two regions in question are located on different arms of chromosome 12. One region is marked by SNP rs76904798 at the 5' end of the *LRRK2* gene on 12q, the other region is highlighted by SNP rs1007709 in close proximity to the promoter region of the *SYT10* gene on 12p. Genetic variation in *LRRK2* is known to be strongly associated with PD risk, and the molecular function of the *LRRK2* gene product is directly related to neural plasticity^25^. Moreover, *SYT10* belongs to the synaptotagmin gene family. Its product, SYT10, can be found in the synapse and is involved in phospholipid binding, syntaxin binding and the cellular response to calcium ion. SYT10 also plays a role in vesicle transport^26^. Worthy of note, the *SYT10* gene belongs to the same gene family as *SYT11*, yet another PD risk gene^4^. The *SYT11* protein mediates calcium-dependent regulation of membrane trafficking in synaptic transmission^27^. Finally, *SYT10* is known to contribute to neuronal function^28^. Current knowledge therefore speaks for a plausible biological interaction between *LRRK2* and *SYT10*.

We identified potential *SYT10* enhancers in brain, located within ±1 Mb from rs1007709, that could potentially modulate the activity of the *SYT10* promoter through differential binding of transcription factors. This modulation may result in altered chromatin structure, changes in gene expression and dysregulation of pathways in which the *SYT10* gene product is involved. SYT10 is a calcium-sensor shown to be required in mice for calcium-dependent exocytosis of secretory vesicles containing IGF1 in neurons of the olfactory bulb, and thus involved in sensory perception of smell^26^. Adding to the plausibility of our findings, PD patients have been shown before to have higher IGF1 levels compared to healthy individuals^29^ and anosmia is a common feature of PD.

The two lead SNPs involved in the most plausible G×G candidate detected, rs76904798 and rs1007709, were found to be only weakly associated in the 1000 Genomes EUR superpopulation (Supplementary Material), suggesting the validity of the independence requirement of the CO design of G×G analysis. Nevertheless, it cannot be excluded that even the small r^2^ estimate of 0.05 obtained from EUR data reflects general long-range LD across the centromere of chromosome 12, or other genes, albeit at low level. This could imply that the interaction OR derived from the IPDGC case data may be an overestimate to a degree depending upon the (unknown) strength of LD in the underlying background population. This reservation notwithstanding, since the association between the two chromosome 12 regions was solitary in the IPDGC data, despite the exceptional power of the data (for a detailed power analysis, see Supplementary Material), it is unlikely to echo a general trend towards cross-centromeric association in the human genome.

In summary, even if our conclusion of statistical G×G interaction between the *LRRK2* and *SYT10* genes was not fully justified, its biological plausibility and independent corroboration by experimental data suggest that the roles of the two genes in PD etiology are functionally linked. Targeted joint studies of the regions, both in vitro and in vivo, may thus help to gain a better understanding of the molecular causes and consequences of PD. Eventually, this may lead not only to more refined means of genetic counseling of *LRRK2* mutation carriers, but also to novel therapeutic or preventive options involving, for example, the interventional modification of *SYT10* gene expression.

## Methods

### SNP genotype data

#### Primary G×G analysis

SNP genotype data used in our primary G×G search originated from the IPDGC and comprised 18,688 cases from 16 different centers in Europe and North America. An overview of the demographic details of all patients is provided in Supplementary Table 9. All human research was approved by the relevant institutional review boards and informed consent was obtained from all human participants. There is a higher proportion of male PD cases across all centers in our study, which is expected as PD is more common in men with a sex ratio of around 2:1 (male:female)^30^. The analysis was first carried out in all cases combined, followed by a subgroup analysis of patients with early onset PD (age at diagnosis <50 years, *n* = 6962).

Depending upon center, genotyping of the IPDGC cases had been undertaken with different types of microarray. All SNP genotype data had been subject to the quality control measures described before^31^. Adopting a threshold of 0.8 for the imputation probability, the total number of SNPs available for further analysis equaled 7.8 million.

Since statistical interaction is equivalent to effect modification, G×G interactions are likely to involve at least one SNP with a known main effect (ME) on disease risk. We therefore focused upon the 90 SNPs reported as disease-associated in the largest PD GWAS to date^4^ and examined all possible pairwise SNP genotype associations between an ME SNP and one of the remaining 7.8 million SNPs.

#### Supportive family cohort data

The data set used to independently support the results of the primary G×G search originated from a family-based cohort of carriers of *LRRK2* mutation p.G2019S (673 PD patients, 339 unaffected individuals), members of which belonged to one of 592 North American, European or Israeli families recruited by The Michael J. Fox Foundation *LRRK2* Consortium or the Tel Aviv Sourasky Medical Center (*LRRK2* family cohort). The institutional review board at each participating site approved the relevant study protocols and informed human consent was obtained from all human participants. These cohorts did not overlap with the IPDGC data. The median age of onset by genotype of the *LRRK2* family cohort can be found in Supplementary Table 10. Further details can be found in the original report of the cohort^21^.

### Statistical analysis

All statistical analyses were performed with either R (v. 3.6.2), PLINK 1.9 or PLINK 2.0^32^.

#### Logistic regression analysis

For our primary G×G search, genotypes *G*_*1*_ of the 90 ME SNPs were encoded according to either a dominant or a recessive model for the minor allele (i.e. *G*_*1*_ = 0 or 1, depending upon genotype). An additive model was additionally considered for genotypes *G*_*2*_ of the remaining SNPs (i.e. *G*_*2*_ = 0, 1 or 2) to ensure coverage of a broad spectrum of possible G×G interactions by our analyses. The logistic regression model of the CO design treated *G*_*1*_ as the response variable and *G*_*2*_ as the predictor variable. With δ_1_ denoting the interaction effect, this leads to regression model as seen in Eq. (1).

1 $${logit}\left\{P\left({G}_{1}=1\right)\right\}=\,{\delta }_{0}+\,{\delta }_{1}{G}_{2}$$

Spurious pair-wise correlation between SNP genotypes may arise when the population under study comprises distinct subpopulations^33^, and genotyping batch effects can create similar artefacts. To address this problem, we carried out center-level principal component analyzes (PCA) of the IPDGC cases with all SNPs that passed quality control and included the top 10 PCs as predictor variables in the logistic regression model. In line with^15^, no classical confounders such as age or biological sex were taken into account. This is because adding a variable to the CO logistic regression model would model interaction between this variable and G_1_, rather than adjust the original G×G interaction of interest for the added variable. Moreover, confounding of a SNP-SNP association in cases, for example, by sex would require (i) that both SNPs show a considerable sex difference in population allele frequency and (ii) that the SNP-SNP association in question is mostly due to this sex difference. Although theoretically possible, such a scenario is highly unlikely for autosomal SNPs.

For the CO design to be valid, G_1_ and G_2_ have to be uncorrelated in the general population^15^. In order to fulfill this requirement, only SNPs on different chromosome arms were considered in the primary G×G search^16^. To allow for possible heterogeneity of the IPDGC data, logistic regression analysis was carried out separately for each center, followed by meta-analysis using a random effects model with inverse variance weights as implemented in R package *metafor*^34^, or using PLINK 2.0, as appropriate. When the minor allele frequency (MAF) of one or both SNPs in a pair is too low, estimates of the logistic regression coefficients can get instable and perturb subsequent meta-analyzes. Therefore, we successively excluded SNP pairs with large confidence intervals for the interaction OR (i.e. for exp(δ_1_)) until stable results were obtained in the meta-analysis. A Wald test was then used to assess whether the average δ_1_, taken over centers, was significantly different from zero. In addition to *p*-values, we report odds ratios with confidence intervals as effect size measures for the G×G interactions studied. Locus zoom plots of chromosomal regions with statistically significant G×G were generated with R, using location information from Ensembl^23^.

To reduce computational and logistic demands, a meta-analysis for a given SNP pair was first conducted considering only those centers that yielded a center-level *p*-value < 0.05 for that pair. All pairs with a meta-analysis *p* < 5 × 10^−5^ were then included in a second meta-analysis comprising all centers. In order to obtain a list of G×G associations with the lowest *p*-values per SNP of interest, 50 G×G associations with the lowest *p*-values per SNP, per center were extracted. Meta-analysis was then performed on these extracted pairs including all possible centers. The five lowest G×G associations per SNP of interest were reported. To control the family-wise error rate, the generally accepted genome-wide *p*-value threshold of 5 × 10^−8^ was Bonferroni-corrected for the 90 possible associations that could arise with one of the ME SNPs, leading to a final *p*-value threshold of 5.6 × 10^−10^ ^35^. No correction for the number of genetic models was undertaken because this would be overly conservative due to the fact that the ensuing statistical test results are strongly correlated.

#### Age-at-onset data analysis

Our analysis of the *LRRK2* family cohort data focused upon those SNPs in the *SYT10* region that achieved *p* < 5.6 × 10^−10^ in the primary G×G search (see Supplementary Material) and that were successfully genotyped in that cohort. The SNPs were assessed for a possible genotypic effect upon the age-at-onset of PD, thus probing interaction not on disease risk per se, but with regard to a PD phenotype. A random effects Cox proportional hazard model was fitted to the age-at-onset data, treating age-at-last visit as a censored observation for unaffected individuals, and treating the appropriately encoded *SYT10* SNP genotype as a predictor variable. Regression coefficients were assessed for statistical significance using a Wald test. All regression models were adjusted for family relationship using a kinship matrix. Since the supportive age-at-onset data analysis was both targeted and confined to a small genomic region with strong local LD, we applied a nominal significance level of 0.05 throughout and abstained from overly conservative multiple testing adjustment.

Since the IPDGC cohort comprised only PD cases (thus no censored observations for age-at-onset), we used a standard linear model to investigate an interaction between selected SNPs on age of onset in cases from the IPDGC cohort as seen in Eq. (2),

2 $$y=\,{G}_{1}+\,{G}_{2}+\,{G}_{1}\times {G}_{2}+{sex}+{{PC}}_{1}+\ldots +{{PC}}_{5}$$

with y being age at onset, G_1_—*SYT10* SNP rs1007709, G_2_—*LRRK2* mutation p.G2019S (rs34637584) or or ME SNP rs76904798 (near LRRK2), sex as a confounder and the first 5 center-level PCs, followed by a meta-analysis (as for the logistic regression analysis).

#### Statistical power

The statistical power to detect G×G with a CO design was calculated using Quanto software^36^. The scenarios considered involved either two SNPs with a low MAF of 0.05 each, or two SNPs with a moderate MAF of 0.2 each. Following Nalls et al. (2020)^4^, the disease OR was set equal to 1.75 for scenario 1, and to 1.25 for scenario 2. The resulting power was determined for sample sizes equal to the total number of cases available in our study (*n* = 18,688), or to 2000. Statistical significance was defined as *p* < 5.6 × 10^−10^. We also calculated the power of a CC design given the same number of cases and an equal number of controls.

#### Post-hoc assessment of SNP associations in controls (1000 genomes)

In an attempt to justify *post hoc* the CO design of our primary G × G search, we ascertained the level of linkage disequilibrium between putatively interacting SNPs in controls. To this end, we used PLINK 2.0 to calculate, from the 1000 Genomes EUR data (*n* = 503), pair-wise allelic association r^2^ and D’ between rs76904798 (in *LRRK2*) and all SNPs with MAF > 0.05 located <1 Mbp upstream of *SYT10* or 1 Mbp downstream of *LRRK2*. To account for possible population differences, *r*^2^ and D’ were calculated separately for each EUR population and a sample size-weighted average was then calculated with R. Moreover, we calculated linkage disequilibrium blocks based on the European population of the 1000 Genomes using default parameters in Plink v. 1.9.

#### Gene expression data analysis

Differences in gene expression between groups of individuals were assessed for statistical significance using either a Wilcoxon test or a Fisher exact test (for dichotomized changes). Expression levels of different genes were related to one another by Pearson correlation coefficients. Since all analyses of the experimental data were exploratory in nature, *p*-values were not corrected for multiple testing.

### Bioinformatics analysis

#### Annotation of regulatory SNP features

Interaction candidate SNPs from the *SYT10* gene region were screened for (i) tissue-specific *SYT10* expression quantitative trait loci (eQTLs) from GTEx Analysis Release V8, (ii) possible regulatory consequences, and (iii) overlaps with, or close vicinity (<50 bp) to, ENCODE *cis*-regulatory elements (CREs)^37^.

#### Chromatin accessibility and histone modifications in brain

H3K27ac and H3K4me1 ChIP-Seq data and corresponding input ChIP-Seq tracks from brain samples were obtained from the CEEHRC (Canadian Epigenetics, Environment and Health Research Consortium) and visualized using the UCSC Genome Browser^38^. ATAC-Seq data from excitatory neurons, interneurons, radial glia and intermediary progenitor cells isolated from mid-gestational samples of the human cortex^39^ were visualized with the WashU EpiGenome Browser^40^.

### In vitro experiments

#### IPSC-derived neurons

Fibroblasts from four PD patients heterozygous for *LRRK2* mutation p.G2019S (G2019S + /PD + ), three unaffected p.G2019S carriers (G2019S + /PD-) and three control individuals lacking a *LRRK2* mutation (G2019S-/PD-) were re-programmed into iPSCs as described^41^. These cell lines were characterized for pluripotency, followed by direct differentiation into dopaminergic neurons as described^42^. Cells from two or three biological replicates were harvested at differentiation days 70 (d70) and 90 (d90) to measure *SYT10* gene expression. During the differentiation process, six of the 60 neuronal cultures detached from the culture plates and were therefore excluded from further analysis. Written informed consent was obtained from all individuals, and the study was approved by the Ethics Committee at the University of Lübeck (approval number 16–039).

#### SYT10 gene expression

RNA was extracted with RNeasy Mini kit (Qiagen, 74106) following the manufacturers’ instructions. cDNA was synthesized using SuperScript^TM^ III reverse transcriptase (Invitrogen, 18080044), with 600 ng of RNA as starting material. PCR was performed using iQ SYBR Green (Biorad, 170–8885). *SYT10* gene expression was normalized to *ACTB* gene expression. Primer sequences used were

*SYT10* forward 5'-GCCTGTTAGCACAAAGAGTGT-3'

*SYT10* reverse 5'-ACAGAACCACCTGCACAACTA-3'

*ACTB* forward 5'-CGAGGACTTTGATTGCACATTGTT-3'

*ACTB* reverse 5'-TGGGGTGGCTTTTAGGATGG-3'.

PCR was run on a LightCycler 480 (Roche). Samples were denatured for 5 min at 95°C. Amplification ran over 45 cycles, with a denaturation step of 10 s at 95°C, primer annealing for 10 s at 60°C, and elongation for 10 s at 72°C.

#### Single-cell gene expression analysis of IPD and control midbrain

We also drew upon previously published single-cell gene expression data from post-mortem midbrain samples from five idiopathic PD cases and six controls (GSE157783). Normalization, sample integration and cell clustering of the data were performed with *Seurat* software (version 3.1.5), as described^43^. For statistical analysis, neuronal clusters were merged and restricted to cells expressing the *SYT10* gene.

### Reporting summary

Further information on research design is available in the Nature Research Reporting Summary linked to this article.

## Supplementary information

Supplementary Material

Reporting Summary

## Data Availability

Participant level data from the IPDGC as used in this study are available to potential collaborators (please contact ipdgc.contact@gmail.com). Aggregate data from the *LRRK2* family cohort are also made available to qualified investigators upon request (dlai@iu.edu). Raw single-cell gene expression data used in our analyses are available at Gene Expression Omnibus (GEO) under accession number GSE157783. Summary data on the *SYT10* expression analysis can be found in Supplementary Table 11. The corresponding data were obtained for this study from the GTEx Portal on 4th June 2021 (dbGaP Accession phs000424.v8.p2).
