# Supplementary material for: Genome-wide case-only analysis of gene-gene interactions with known Parkinson’s disease risk variants reveals link between LRRK2 and SYT10
Source: NPJ Parkinsons Dis. 2023 Jun 29;9:102. doi: 10.1038/s41531-023-00550-9 (PMC10310744; doi:10.1038/s41531-023-00550-9)
Supplement: Supplementary file 2 — Reporting Summary [file 41531_2023_550_MOESM2_ESM.pdf]

nature portfolio

Corresponding author(s): Prof. Dr. Astrid Dempfle

Last updated by author(s): Feb 15, 2023

Reporting Summary

Nature Portfolio wishes to improve the reproducibility of the work that we publish. This form provides structure for consistency and transparency in reporting. For further information on Nature Portfolio policies, see our Editorial Policies and the Editorial Policy Checklist.

Statistics

For all statistical analyses, confirm that the following items are present in the figure legend, table legend, main text, or Methods section.

n/a	Confirmed	
☐	☒	The exact sample size (*n*) for each experimental group/condition, given as a discrete number and unit of measurement
☐	☒	A statement on whether measurements were taken from distinct samples or whether the same sample was measured repeatedly
☐	☒	The statistical test(s) used AND whether they are one- or two-sided<br>*Only common tests should be described solely by name; describe more complex techniques in the Methods section.*
☐	☒	A description of all covariates tested
☐	☒	A description of any assumptions or corrections, such as tests of normality and adjustment for multiple comparisons
☐	☒	A full description of the statistical parameters including central tendency (e.g. means) or other basic estimates (e.g. regression coefficient) AND variation (e.g. standard deviation) or associated estimates of uncertainty (e.g. confidence intervals)
☐	☒	For null hypothesis testing, the test statistic (e.g. *F*, *t*, *r*) with confidence intervals, effect sizes, degrees of freedom and *P* value noted<br>*Give P values as exact values whenever suitable.*
☒	☐	For Bayesian analysis, information on the choice of priors and Markov chain Monte Carlo settings
☒	☐	For hierarchical and complex designs, identification of the appropriate level for tests and full reporting of outcomes
☐	☒	Estimates of effect sizes (e.g. Cohen's *d*, Pearson's *r*), indicating how they were calculated

Our web collection on statistics for biologists contains articles on many of the points above.

Software and code

Policy information about availability of computer code

Data collection	For the main analysis, genotyping was conducted using the 650Y, Human660-Quad, Human610K, HumanCNV370 version1_C, HumanHap300 or Infinium BeadChips (Illumina). data were imputed using Michigan Imputation Server, using the Haplotype Reference Consortium data as the imputation base, threshold of 0.8 for the imputation probability and otherwise using default settings.<br>GTEx Analysis Release V8 was used for tissue-specific SYT10 expression quantitative trait loci (eQTLs).<br>H3K27ac and H3K4me1 ChIP-Seq data and corresponding input ChIP-Seq tracks from brain samples were obtained from the CEEHRC (Canadian Epigenetics, Environment and Health Research Consortium).<br>RNA was extracted with RNeasy Mini kit (Qiagen, 74106) following the manufacturers' instructions. cDNA was synthesized using SuperScriptTM III reverse transcriptase (Invitrogen, 18080044), with 600 ng of RNA as starting material. PCR was performed using iQ SYBR Green (Biorad, 170-8885). PCR was run on a LightCycler 480 (Roche).
Data analysis	All statistical analyses were performed with either R (v. 3.6.2), PLINK 1.9 or PLINK 2.0.<br>H3K27ac and H3K4me1 ChIP-Seq data and corresponding input ChIP-Seq tracks from brain samples were visualized using the UCSC Genome Browser.<br>ATAC-Seq data from excitatory neurons, interneurons, radial glia and intermediary progenitor cells isolated from mid-gestational samples of the human cortex were visualized with the WashU EpiGenome Browser.<br>Normalization, sample integration and cell clustering of the data were performed with Seurat software (version 3.1.5).

For manuscripts utilizing custom algorithms or software that are central to the research but not yet described in published literature, software must be made available to editors and reviewers. We strongly encourage code deposition in a community repository (e.g. GitHub). See the Nature Portfolio guidelines for submitting code & software for further information.

Data

Policy information about availability of data

All manuscripts must include a data availability statement. This statement should provide the following information, where applicable:

- Accession codes, unique identifiers, or web links for publicly available datasets
- A description of any restrictions on data availability
- For clinical datasets or third party data, please ensure that the statement adheres to our policy

Data not generated for this study (and already used in previous publications): Participant level data from the IPDGC as used in this study are available to potential collaborators (please contact ipdgc.contact@gmail.com). Aggregate data from the LRRK2 family cohort are also made available to qualified investigators upon request (dlai@iu.edu).
Data specifically generated for this study: Raw single-cell gene expression data used in our analyses are available at Gene Expression Omnibus (GEO) under accession number GSE157783. Summary data on the SYT10 expression analysis can be found in Supplementary Table 6.

Field-specific reporting

Please select the one below that is the best fit for your research. If you are not sure, read the appropriate sections before making your selection.

☒ Life sciences ☐ Behavioural & social sciences ☐ Ecological, evolutionary & environmental sciences

For a reference copy of the document with all sections, see nature.com/documents/nr-reporting-summary-flat.pdf

Life sciences study design

All studies must disclose on these points even when the disclosure is negative.

Sample size	Primary G×G analysis: 18 688 cases from 16 different centers. To the best of our knowledge, this is the largest available dataset for PD cases. A statistical power analysis was done (see Supplement for more details) indicating, that, given the case-only study design, this sample size will provide ≥80% power for an interaction OR >1.25 at MAF=0.2, and for OR >1.55 at MAF=0.05. The case-only design provided consistently greater statistical power than a case-control design given the same number of cases.
Data exclusions	In order to fulfill the assumption of independence necessary for the case-only study design, only SNPs on different chromosome arms were considered in our primary G×G search. When the minor allele frequency (MAF) of one or both SNPs in a pair is too low, estimates of the logistic regression coefficients can get instable and perturb subsequent meta-analyses. Therefore, we successively excluded SNP pairs with large confidence intervals for the interaction OR (i.e. for exp(δ1)) until stable results were obtained in the meta-analysis. Moreover, to reduce computational and logistic demands, a meta-analysis for a given SNP pair was first conducted considering only those centres that yielded a centre-level p value <0.05 for that pair. All pairs with a meta-analysis p<5×10-5 were then included in a second meta-analysis comprising all centres.
Replication	Support of any putative G×G interactions found was sought by the analysis of independent genotype-phenotype and experimental data. SNPs around SYT10 were also associated with the age-at-onset of PD in an independent cohort of carriers of LRRK2 mutation p.G2019S. Moreover, SYT10 gene expression during neuronal development was found to differ between cells from affected and non-affected p.G2019S carriers.
Randomization	Randomization was not relevant for our type of analysis.
Blinding	Blinding was not relevant for our type of analysis.

Reporting for specific materials, systems and methods

We require information from authors about some types of materials, experimental systems and methods used in many studies. Here, indicate whether each material, system or method listed is relevant to your study. If you are not sure if a list item applies to your research, read the appropriate section before selecting a response.

Materials & experimental systems

n/a	Involved in the study	
☒	☐	Antibodies
☒	☐	Eukaryotic cell lines
☒	☐	Palaeontology and archaeology
☒	☐	Animals and other organisms
☐	☒	Human research participants
☒	☐	Clinical data
☒	☐	Dual use research of concern

Methods

n/a	Involved in the study	
☒	☐	ChIP-seq
☒	☐	Flow cytometry
☒	☐	MRI-based neuroimaging

Human research participants

Policy information about studies involving human research participants

Population characteristics	Primary G×G analysis: 18 688 cases from 16 different centers. Logistic regression was carried out on each center separately and results were combined by meta-analysis using a random effects model with inverse variance weights. Centre-level principal component analyses of the IPDGC cases with all SNPs that passed quality control was carried out and the top 10 principal components were included as predictor variables in the logistic regression model. Since a case only study design was applied, in line with Piegorsch et al. (1994) (see reference 14), no classical confounders such as age or biological sex were taken into account.
Recruitment	Bias is reduced by applying the case-only study design - since only cases are needed, there are no issues with appropriate selection of controls. Recruitment was done in each of the 16 centers independently.
Ethics oversight	Local ethics approval was obtained by each participating center separately. No human research participant were recruited specifically for this study, only genotype data generated previously was used for a new statistical analysis, therefore no new ethics approval was necessary.

Note that full information on the approval of the study protocol must also be provided in the manuscript.
